# Activity-based cell sorting reveals responses of uncultured archaea and bacteria to substrate amendment

**DOI:** 10.1038/s41396-020-00749-1

**Published:** 2020-09-04

**Authors:** Nicholas J. Reichart, Zackary J. Jay, Viola Krukenberg, Albert E. Parker, Rachel L. Spietz, Roland Hatzenpichler

**Affiliations:** 1 Department of Chemistry and Biochemistry, Montana State University, Bozeman, MT 59717 USA; 2 Thermal Biology Institute, Montana State University, Bozeman, MT 59717 USA; 3 Center for Biofilm Engineering, Montana State University, Bozeman, MT 59717 USA; 4 Department of Mathematical Sciences, Montana State University, Bozeman, MT 59717 USA

**Keywords:** Environmental microbiology, Microbial communities, Microbial ecology, Archaea

## Abstract

Metagenomic studies have revolutionized our understanding of the metabolic potential of uncultured microorganisms in various ecosystems. However, many of these genomic predictions have yet to be experimentally tested, and the functional expression of genomic potential often remains unaddressed. In order to obtain a more thorough understanding of cell physiology, novel techniques capable of testing microbial metabolism under close to in situ conditions must be developed. Here, we provide a benchmark study to demonstrate that bioorthogonal non-canonical amino acid tagging (BONCAT) in combination with fluorescence-activated cell sorting (FACS) and 16S rRNA gene sequencing can be used to identify anabolically active members of a microbial community incubated in the presence of various growth substrates or under changing physicochemical conditions. We applied this approach to a hot spring sediment microbiome from Yellowstone National Park (Wyoming, USA) and identified several microbes that changed their activity levels in response to substrate addition, including uncultured members of the phyla *Thaumarchaeota*, *Acidobacteria*, and *Fervidibacteria*. Because shifts in activity in response to substrate amendment or headspace changes are indicative of microbial preferences for particular growth conditions, results from this and future BONCAT-FACS studies could inform the development of cultivation media to specifically enrich uncultured microbes. Most importantly, BONCAT-FACS is capable of providing information on the physiology of uncultured organisms at as close to in situ conditions as experimentally possible.

## Introduction

Traditionally, metagenomic predictions about the ecophysiology of microbes have often been tested using stable isotope probing (SIP), which can determine cellular activity through incorporation of growth substrates. SIP experiments have been limited by their need for isotope-labeled molecules that can be prohibitively expensive or unavailable because of compositional complexity. An alternative to SIP targeted at a specific assimilatory pathway is to label all anabolically active cells in a sample with deuterated water (^2^H_2_O) and visualize heavy isotope uptake into individual cells using Raman microspectroscopy [1, 2]. Laser tweezers can then be used to separate functionally active cells from the microbial community [1, 2]. However, even using automated platforms, Raman-activated cell sorting currently can only achieve sorting rates of up to 500 cells/h [3]. Such low-throughput limits functional tests to the most abundant, active members of a community, and a handful of incubation conditions of high interest to the researcher.

Bioorthogonal non-canonical amino acid tagging combined with fluorescence-activated cell sorting (BONCAT-FACS) is a recently developed Next-Generation Physiology approach [4] that can be used to identify uncultured microorganisms that are translationally active at a rate of thousands of cells per second [5–9]. BONCAT tracks translational activity through the incorporation of a synthetic amino acid, such as *L*-homopropargylglycine (HPG). HPG is a structural analog of *L*-methionine and is incorporated into newly synthesized proteins due to the substrate promiscuity of methionyl-tRNA synthetase [10]. HPG-containing proteins can be detected via copper-catalyzed azide-alkyne click chemistry dye staining and subsequently visualized with epifluorescence microscopy [5, 6, 11]. In combination with FACS, BONCAT fluorescence can be used to sort translationally active members of a microbial community and taxonomically identify them with subsequent 16S ribosomal RNA (rRNA) gene sequencing [6, 7]. BONCAT-FACS was recently used to identify the anabolically active fraction of microbes in soil [7] and deep-sea sediments [6] under close to in situ conditions. A previous study demonstrated the capability of BONCAT to reveal the activity response of cultured bacteria to substrate amendment [5], suggesting that BONCAT could also be used to identify beneficial growth conditions for yet uncultured microorganisms. Here, we used a sediment slurry sample from a hot spring in Yellowstone National Park (YNP) to demonstrate that BONCAT-FACS can be used to test genomic predictions of microbial physiology and screen community-wide responses in anabolic activity to numerous treatments in parallel, including amendments with potential growth substrates as well as varied headspace conditions.

## Materials and methods

### Incubation setup

**Fig. 1:**
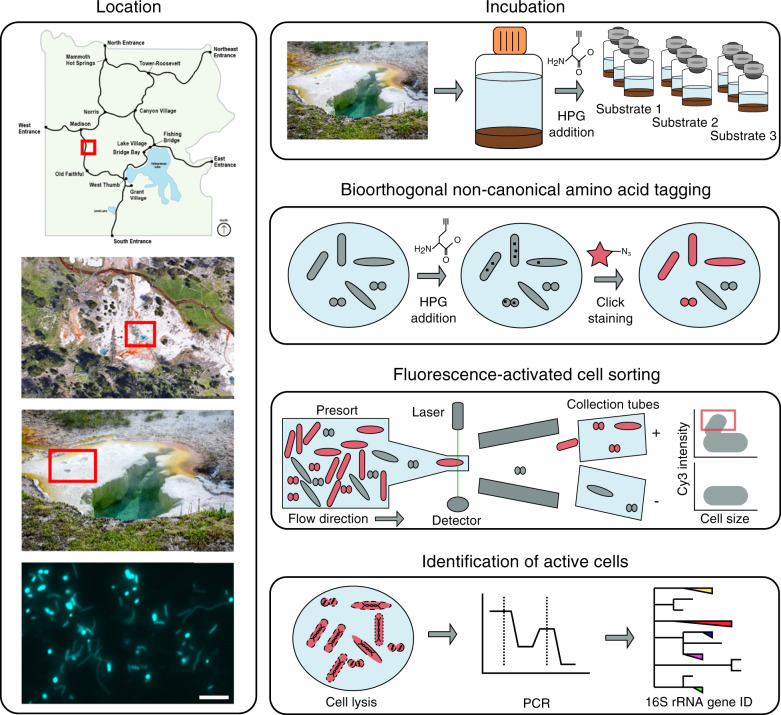
Site location and workflow for BONCAT-FACS identification of active cells from environmental samples. Location of hot spring FS5 in YNP. Microscopy image of unsorted hot spring cells visualized with general nucleic acid stain, 4′,6-diamidino-2-phenylindole (DAPI). Scale bar represents 5 µm. Samples were incubated in the presence of *L*-homopropargylglycine (HPG) under various treatments (Supplementary Table 1) for 48 h. Cells were detached from particle material and newly synthesized proteins were dye-stained via azide-alkyne click chemistry. Translationally active, fluorescent cells were sorted using fluorescence-activated cell sorting (FACS) from the presort, total extractable cell community. Cells were lysed and 16S rRNA genes were amplified with PCR. Black squares (■) in cells represent new proteins containing HPG. Map of YNP provided by the US National Park Service. Satellite image obtained from Google Maps.

All work in YNP was conducted under US National Park Service permit no. YELL-SCI-8010 (2017–2019). A sediment slurry sample (~10% sediment, 90% water) was collected using a stainless-steel measuring cup fixed to the end of a 12-foot telescoping pole from a hot spring within the Five Sisters hot spring group in YNP (44.532619, −110.797272) (Fig. 1). At the time of sampling the temperature, pH, and dissolved oxygen of the spring were 72.2 °C, 8.45, and 0.909 mg/L (Hach DR900 dissolved oxygen colorimetric assay), respectively. The slurry sample was transferred to a 1 L acid-washed and autoclaved glass bottle, sealed with a screw cap, and transported to the laboratory in an insulated heated container within 4 h of sampling, maintaining an ambient temperature of 55 °C. Incubation experiments were prepared the day following sample collection after allowing the sample to adjust to 74 °C in an incubator overnight. Each incubation consisted of 6 mL homogenized slurry aliquoted into 30 mL acid-washed and autoclaved glass vials. The slurry was kept homogenous by stirring with a magnetic stir bar during setup. A T_0_ sample was taken from the homogenous mixture and frozen in 10% glycerol-TE [12] buffer (glycerol, Tris-EDTA buffer, milliQ water) for later DNA extraction. A set of three vials to which no substrate or bioorthogonal amino acid was added served as a negative control (i.e., no-HPG control). After the sample was transferred to the no-HPG control, *L*-homopropargylglycine (HPG; Click Chemistry Tools) was added to the remaining bulk slurry to yield a final concentration of 50 µM. Afterwards, 6 mL of the HPG-containing slurry was transferred to all incubation vials that had already been amended with a substrate (Supplementary Table 1). Substrates were confirmed to be stable at 74 °C for 48 h by liquid chromatography mass spectrometry (LC–MS) analysis (not shown). A set of three vials, which contained no substrate amendment but HPG, served as a control (i.e., HPG-only control) for close to in situ activity of the microbial community. All vials were sealed with sterile butyl rubber stoppers with ambient lab air as a headspace (21% O_2_). Two sets of triplicate incubations tested headspace composition on microbial activity, 2% O_2_ (microoxic), and 100% N_2_ (anoxic) were prepared by flushing the headspace with 0.2 µm filtered N_2_ gas for 3 min. The vials for 2% O_2_ incubations had ~10% of the N_2_ headspace volume removed and replaced with 0.2 µm filtered ambient lab air. All incubations were performed in triplicate and were incubated at 74 °C in the dark for 48 h without shaking. This incubation time was chosen after preliminary experiments had been performed with 6, 24, and 48 h incubation times. An incubation time of 48 h was selected to increase cell labeling rates to allow for the reliable sorting of BONCAT-positive cells. At the completion of the incubation, vials were uncapped, and the content of each vial was immediately transferred to a 15 mL conical tube with 10% glycerol-TE [12] buffer (glycerol, Tris-EDTA buffer, milliQ water) to preserve cells. Afterwards, these tubes were stored at −80 °C until further processing.

### Sample processing

For cell extractions, samples preserved in glycerol-TE were thawed at 4 °C, vortexed briefly at maximum speed, and 1 mL of slurry were transferred to a 15 mL conical tube containing 4 mL of 1× phosphate buffered saline (PBS) with Tween 20 (Promega) at a final concentration of 0.01%. The conical tubes were vortexed for 5 min at maximum speed to detach cells from particles before being centrifuged for 5 min at 500 × *g* to separate particles from the biomass. Following centrifugation, the cell-containing supernatant was passed through a 35 µm pore size filter and the filtrate was divided evenly across 1.5 mL tubes and centrifuged at 14,000 × *g* for 5 min to pellet the cells. The supernatant was discarded by careful pipetting, and the pellet of each sample was resuspended and combined in a final volume of 300 µL 1× PBS. A bulk click reaction solution was prepared [11] and 200 µL of this mix was aliquoted to each sample. Succinctly, the final reaction mix was comprised of 5 mM amino guanidine hydrochloride (Sigma Aldrich), 5 mM sodium *L*-ascorbate (Sigma Aldrich), 100 µM copper sulfate pentahydrate (Sigma Aldrich), 500 µM THPTA (Click Chemistry Tools), and 4 µM Cy3 picolyl-azide dye (Click Chemistry Tools) in 1× PBS. The reaction mixtures were vortexed briefly to mix and then rotated in the dark at room temperature for 1 h. After this incubation step, all samples were washed three times by a series of centrifugation steps at 14,000 × *g* for 5 min and resuspended in 1 mL of 1× PBS. Following the final wash, cells were resuspended in 500 µL of 1× PBS and stored at 4 °C in the dark before being sorted on a fluorescence-activated cell sorter (FACS) the subsequent day. One replicate of each incubation condition was extracted per day and all samples were checked for successful completion of the click reaction by epifluorescence microscopy (Leica DM4B microscope with DAPI and Cy3 filter sets).

### Fluorescence-activated cell sorting

On the day following cell extraction and click staining, cells were sorted based on Cy3 fluorescence intensity using a Sony SH800S FACS. Prior to sorting, a 50 µL aliquot of each sample (“Presort” sample) was transferred to a new tube and stored at 4 °C for later processing. For sorting, a first gate was drawn based on forward scatter area (FSC-A) and back scatter area (BSC-A) to exclude any large particles that remained after filtration (Supplementary Fig. 1). A second gate was then drawn based on forward scatter width (FSC-W) and forward scatter height (FSC-H) to further constrain the size of particles examined. The third and final gate was determined using FSC-A and Cy3 fluorescence intensity to capture fluorescent, translationally active (“BONCAT gate”) cells only. The gate for Cy3 fluorescence was drawn based on the no-HPG control which contained only background fluorescence of the sample and no positive BONCAT signal. The fluorescence of the no-HPG control was recorded to define the lower limit of the “BONCAT gate” to capture <0.1% of all events in the no-HPG control (Supplementary Fig. 1). The HPG-only control was also recorded to define the upper limits of the gate that captured fluorescence cells. Cells within the “BONCAT gate” were sorted into 1.5 mL tubes containing 200 µL of 1× PBS to prevent cells from sticking to the tube walls and lysing. The cell sorter contained sheath fluid composed of UV-sterilized and 0.2 µm filtered 1× PBS. Each sample was sorted for a maximum of 250,000 fluorescent events or until the volume was exhausted prior to meeting this total (see Supplementary Table 2 for a list of events sorted per replicate). Sorted cells were stored at 4 °C. At the end of each day of FACS sorting, all samples, including “Presort” samples, from that day were centrifuged at 14,000 × *g* for 5 min and the supernatants were discarded by careful aspiration before the cell pellets were resuspended in 20 µL of nuclease-free water. Epifluorescence microscopy was used to check the purity of sorted cells, confirming the presence of only fluorescent cells in the “BONCAT gate” (not shown). Cells were stored at 4 °C until all samples were sorted. SYBR Green I nucleic acid stain (Invitrogen) was tested as a counterstain to fluorescently identify all events containing biomass prior to gating on a BONCAT fluorescent signal. However, after testing several different combinations of SYBR Green I with clickable dyes (i.e., Cy3, 594, and Cy5) it was determined that too much bleed-over across fluorescence channels would prohibit the reliable sorting of SYBR and BONCAT-positive events with the current laser and detector setup.

### DNA extraction, PCR, and sequencing

For DNA extraction, three cycles of freeze–thaw were used to lyse cells. Cells stored in 20 µL nuclease-free water at 4 °C were transferred into 96-well microtiter plates that were sealed with sterile adhesive foil sheets. Freezing was performed by placing the plate into a −80 °C freezer for 20 min. Thawing was done at 99 °C for 10 min in a thermal cycler (Eppendorf Mastercycler nexus GSX1). Prior to each subsequent freezing step, the plates were pulse centrifuged at 500 × *g*. The T_0_ sample taken at the beginning of the incubation was extracted using the FAST DNA spin kit for soil (MP Biomedicals). Amplification of bacterial and archaeal 16S rRNA genes was performed by PCR using the Earth Microbiome protocol [13] with updated 515F [14] (5′-GTGYCAGCMGCCGCGGTAA-3′) and 806R [15] (5′-GGACTACNVGGGTWTCTAAT-3′) primers directly into the microtiter plates containing the extracted DNA. The final PCR volume was 50 µL and consisted of 20 µL Invitrogen Platinum Taq II 2X Master Mix, 1 µL 515F primer (10 µM; final: 0.2 µM), 1 µL 806R primer (10 µM; final: 0.2 µM), and 8 µL nuclease-free water added directly into the microtiter plates containing the 20 µL of lysate. The thermocycler conditions were: 94 °C for 3 min followed by 28 cycles of 94 °C for 45 s, 50 °C for 60 s, and 72 °C for 90 s before a final elongation step at 72 °C for 10 min. PCR products were purified with Ampure XP beads (Beckman Coulter) to remove excess primers following the manufacturer’s protocol with a final elution step in 40 µL nuclease-free water. A second PCR reaction was performed to attach dual barcode indices and sequencing adapters to the previously amplified and purified products. This PCR was performed in a 25 µL final volume with 5 µL purified, amplified DNA, 12.5 µL Invitrogen Platinum Taq II 2X Master Mix, 2.5 µL i5 primer (final: 0.25 µM), 2.5 µL i7 primer (final: 0.25 µM), and 2.5 µL water. The PCR conditions were as follows: 95 °C for 3 min followed by 8 cycles of 95 °C for 30 s, 55 °C for 30 s, and 72 °C for 30 s, followed by a final elongation step at 72 °C for 5 min. Following completion of the barcoding PCR, samples were randomly selected and checked for presence of a PCR band on a 1.5% agarose gel. From each PCR plate, a no template control and a positive control that used *Escherichia coli* (*E. coli*) DNA were included. After confirming correct band length, each PCR reaction was again purified with Ampure XP beads to remove excess primers. Triplicate Quant-iT Picogreen dsDNA Assay (Invitrogen) reactions were performed for all samples following manufacturer’s guidelines and quantified with a Biotek Synergy H1 Hybrid microplate reader. For each sample, excluding *E. coli* positive controls, 10 ng of barcoded and cleaned DNA per sample was pooled. Additional samples that were amplified and included in the sample pool were reagent extraction blanks and sheath fluid aliquots from the sorter sheath fluid tank. Half of the pooled volume was concentrated with the QIAquick PCR purification spin column kit (Qiagen) following manufacturer’s guidelines. Sequencing was performed by Laragen Inc. (Culver City, CA) using Illumina 2 × 250 paired end read MiSeq sequencing. A total of 12,071,436 raw reads were generated from 209 samples.

### Testing of FACS sorting and DNA extraction efficiency

The minimum number of sorted events required for sufficient biomass recovery of an organism was determined by sorting events from an *Aliivibrio fischeri* pure culture, and sorting purity was confirmed by 16S rRNA gene sequencing. Events ranging from 5 × 10^2^ to 5 × 10^4^ were sorted and sequenced. It was determined that *A. fischeri* accounted for 98.91% of the reads when 5 × 10^4^ events were sorted. The sequencing reads assigned to *A. fischeri* decreased with decreasing number of events sorted, consistent with literature reports on FACS [16]. Therefore, 5 × 10^4^ events sorted was used as a minimum threshold of events for sample processing.

Validation of successful DNA extraction through consecutive freeze–thaw cycles was confirmed with four pure culture isolates and one environmental sample. The following cultures were purchased from the German Collection of Microorganisms (DSMZ) and grown in the recommended media: *Caldicellulosiruptor bescii* (DSMZ 6725), *Desulfobacterium autotrophicum* (DSMZ 3382), *Rhodopirellula baltica* (DSMZ 10527), and *Aliivibrio fischeri* (DSMZ 507). The environmental sample tested was sediment-water slurry from hot spring FS5. DNA was quantified by Qubit high sensitivity dsDNA assay (Invitrogen) on varying amounts of cell numbers (achieved by consecutive 1:10 dilutions) for each test sample. The lowest quantity of cells required for detection with this Qubit assay was determined to be 100,000 cells for *R. baltica* while all other cultures needed a minimum of 250,000 cells. DNA was extracted from FS5 with the FAST DNA spin kit for soil (MP Biomedicals) and 16S rRNA genes were amplified and sequenced as described above. The community composition of the environmental sample based on 16S rRNA gene sequence analysis was comparable to the sequence composition of the “presort” samples that were lysed with repeated freeze–thaw cycles. Sequence composition from the DNA extraction kit samples were similar to the composition of samples extracted with freeze–thaw cycles.

### BONCAT substrate specificity tests with *Escherichia coli*

*E. coli* was grown overnight in an Erlenmeyer flask in M9 minimal media with 0.04% glucose. The culture was then split into equal volumes and received new M9 media as before but containing different substrate amendments. Five conditions were tested in triplicate with the following addition to the base medium (0.04% glucose) and a final concentration of 50 µM HPG; 0.36% sucrose, 0.36% sorbitol, 0.36% glucose, 0.36% glucose and 10 µg/mL chloramphenicol, or no substrate addition. A sixth condition was tested without additional substrate or HPG. After 2 h of incubation, *E. coli* cells were fixed with 2% paraformaldehyde for 1 h at room temperature, washed three times with 1× PBS, and click stained with Cy3 picolyl-azide dye. Click stained cells were visualized with an epifluorescence microscope and imaged with Leica software. Images were analyzed using daime software package [17] for relative fluorescence units (RFU). RFU were averaged for each condition (Supplementary Fig. 2).

### Amplicon data processing

Sequence reads were processed using QIIME 2 (version 2018.6) [18]. Briefly, barcode adapter sequences were first removed using cutadapt prior to truncation of the forward (130 bases) and reverse (150 bases) reads based on review of the sequencing quality. Reads were then denoised, merged, and chimera-checked with DADA2, resulting in 4627 amplicon sequence variants (ASVs). Taxonomy was assigned using Silva SSU database release 128 with the classify-sklearn method. R [19] package *decontam* (version 1.1.2) [20] was run to remove contaminants using the “Prevalence” model with a threshold of 0.5. *decontam* identified 438 contaminating ASVs which represented 399,454 reads (3.31% of total reads). Samples represented by at least 5000 sequence reads were used for further analysis. ASVs were collapsed at the genus level, relative sequence abundance was calculated for each sample, and only taxa above 0.01% across all samples were analyzed. From here on, relative abundance is referring to the relative abundance of sequencing reads. Outlying replicates were determined using the *DESeq2* package [21] by clustering rlog transformed counts by Euclidean distance and clustering raw counts by Poisson distance. One presort sample was removed as an outlier (Glycine replicate 3) and four sorted samples were removed (Biotin replicate 3, Leucine replicate 3, Glycine replicate 1, and Isoleucine replicate 2). Removed samples were confirmed by manual inspection of sequence data and all samples contained a majority of reads attributed to known contaminating organisms that were not filtered with *decontam*. Microbial community analysis was performed in R [19] using PERMANOVA with substrate and replicate as factors in the vegan package [22] with Bray–Curtis dissimilatory. Shannon’s diversity index was calculated for all treatments and separately compared to the HPG-only controls using linear mixed effects models in the *lme4* package [23] with substrate treatment as a fixed effect and replicate as a random effect with *p* values adjusted for multiple comparisons [24]. Log2-fold change (LFC) of the taxa expression in each treatment was assessed using a negative binomial model with treatment and replicate as factors in *DESeq2*. All sequence data associated with this study have been deposited in *Genbank* under BioProject number PRJNA601738. All files generated during data processing are available in the Supplementary Online Information.

## Results and discussion

To expand on previous BONCAT findings, we initially performed an experiment with *E. coli*, which demonstrated that translational activity as measured by BONCAT can only be detected when cultures were grown with glucose or sorbitol as the sole carbon and energy source but not with sucrose (Supplementary Fig. 1). This is consistent with the notion that *E. coli* is genetically incapable of sucrose utilization. Addition of chloramphenicol, an antibiotic targeting ribosome function, to the culture decreased the BONCAT fluorescence intensity to background levels, i.e., fluorescence values observed when cells were grown with HPG but without growth substrate. These results demonstrated that BONCAT can be used to study cell activity responses to substrate amendment and suggested that it could be used to study complex microbiomes.

**Fig. 2:**
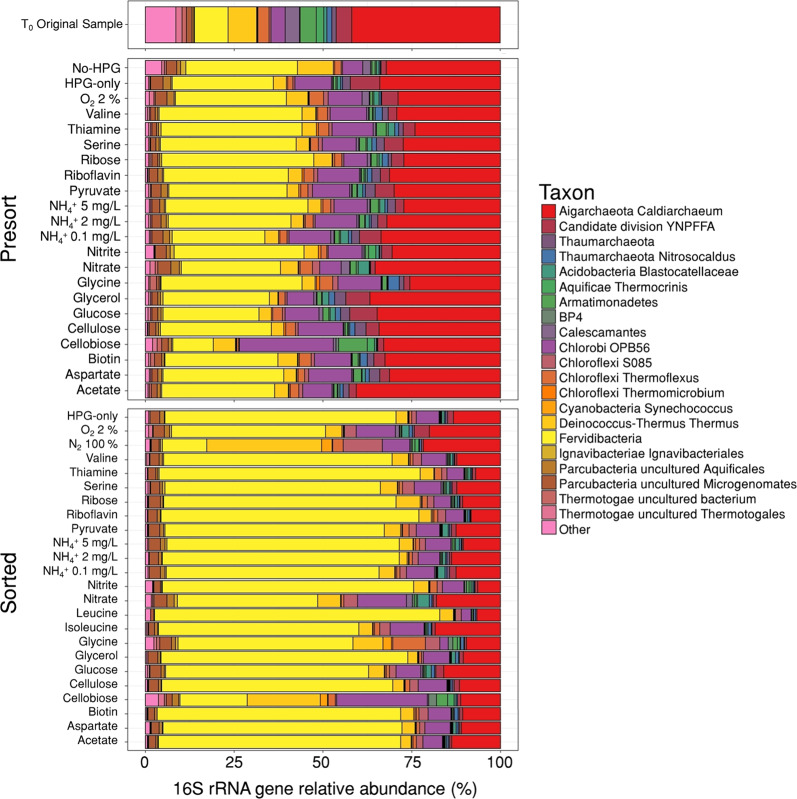
16S rRNA gene relative abundances in averaged incubations. Top panel, unamended FS5 community at the time of sampling (T_0_). Middle panel, community composition of presort samples, representing the extractable microbial community, after the incubation experiments. Bottom panel, composition of the sorted active cell fraction after incubation with substrate amendment. Only taxa that were represented above 1% in at least one sample are shown. Additional taxa were combined into “Other” category. Taxon level represents highest taxonomic resolution for each taxa (Silva 128). Some replicates did not pass the quality control steps of our bioinformatics pipeline (<5000 reads), which precluded including them in this figure.

For this benchmark study, we selected a high temperature (74 °C), alkaline (pH 8.2) hot spring (Five Sisters 5, FS5) in the Lower Geyser Basin of YNP (Fig. 1). Hot springs harbor lower complexity microbial communities compared to other environments, such as soils and marine sediments, making them an ideal system for the development of novel approaches. As indicated by 16S rRNA gene amplicon sequencing, hot spring FS5 was dominated by the archaeal candidate phylum *Aigarchaeota* (41.9%), with the next most abundant community members belonging to bacterial candidate phylum *Fervidibacteria* (9.57%) and phylum *Deinococcus-Thermus* (7.98%) (Fig. 2). Members of the *Aigarchaeota* have been detected in many high temperature (65–88 °C) hot springs over a wide pH range (2.9–9.3) [25]. Despite multiple studies describing their versatile metabolic potential, *Aigarchaeota* remain recalcitrant to cultivation and no experimental data on their functional activity are currently available. Reconstructed genomes suggest the capacity for aerobic respiration or anaerobic respiration with nitrate as an electron acceptor [25]. In addition, a recent study found multiple endoglucanases and β-glucosidases that might be involved with degradation of cellulose and cellobiose in an *Aigarchaeota* metagenome bin [26]. One of the goals of our study was to test these functional predictions using BONCAT-FACS.

We applied BONCAT-FACS combined with 16S rRNA gene sequencing to identify the active microbial community members in hot spring material when incubated in the presence of 23 different substrates or growth conditions (Fig. 1; see list of treatments in Supplementary Table 1). Because BONCAT relies on the incorporation of a synthetic amino acid as an indirect activity tracer, we were also able to test activity responses toward changes of headspace gas composition. Specifically, we tested the activity of microbes in the presence of atmospheric (21% O_2_), microoxic (2% O_2_), and anoxic (100% N_2_) headspace conditions. Laboratory incubations were established with a slurry containing FS5 water and sediment to which HPG and substrates were added. After 48 h of incubation, biomass was resuspended in glycerol Tris-EDTA buffer [12] and samples were frozen at −80 °C for later processing. After samples were thawed, cells were extracted from the slurry, fluorescently stained using click chemistry, and sorted via FACS based on fluorescence signal. The sorted cells were lysed with repeated freeze–thaw cycles prior to PCR amplification of their 16S rRNA genes. An HPG-only incubation was used to identify cells active under close to in situ conditions (with low perturbation) and represented the baseline for comparing cellular activity under varying incubation conditions.

The microbial community of FS5 was highly active as represented by the high proportion of BONCAT-labeled events in the HPG-only control (Supplementary Table 1). Furthermore, all abundant taxa (>1% relative abundance of the total extractable community, ASVs collapsed to the genus level) were active in at least one condition, and most taxa were active under several conditions (Fig. 2). This demonstrates that BONCAT can be applied to a wide variety of phylogenetic groups, which is consistent with previous reports [5–9, 27]. The sorted, active fraction from incubations with different treatments contained no statistically significant difference when compared to the HPG-only control based on Bray–Curtis dissimilarity (MANOVA, *p* = 1) (Fig. 2). This result indicates that the microbial activity response to any treatment, as captured by BONCAT, was not large enough or consistent enough among replicates to significantly change the overall active community when compared to the HPG-only control.

**Fig. 3:**
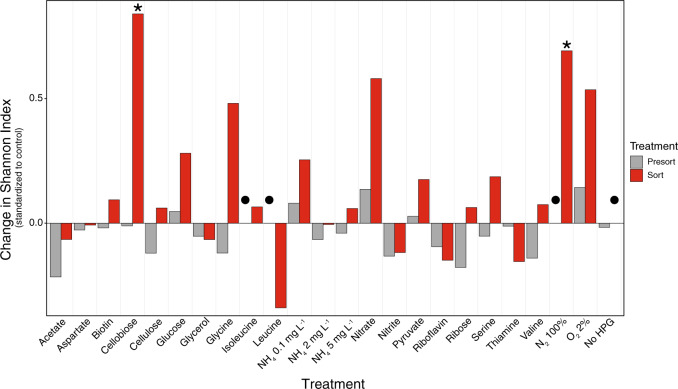
Change in Shannon’s diversity indices of each treatment standardized to HPG-only controls. The Shannon’s diversity indices for presort and sorted samples were compared relative to their respective HPG-only controls. The value for HPG-only was set to 0 and the difference for each sample is plotted. Overall, presort samples exhibited less variability than sorted samples. Sorted samples for cellobiose and anoxic conditions (100% N_2_ headspace) were significantly different from the sorted HPG-only (atmospheric air) control (*p* < 0.05) and marked with an asterisk (*). Samples marked with a circle (●) indicate no data available.

Although the overall composition of the active communities did not vary significantly in response to incubation conditions, changes within the richness and evenness of the communities were detected. To further describe the community composition, the Shannon’s diversity index of each incubation was calculated (Fig. 3). For each treatment, the bulk, presort fraction (representing the total, extractable cell community), and the sorted, active cell fraction were analyzed and compared to the respective HPG-only control. Overall, the variation in the presort fraction of Shannon’s diversity indices was less variable than the sorted fraction from the same treatment incubation (Fig. 3). This demonstrated that the BONCAT-FACS approach could detect changes in the diversity of the active community due to individual cell responses prior to shifts in cell abundance occurring in presort communities. We expected little variation in the presort community because the limited time of incubation should not have allowed for an overall shift in community composition on a bulk level. However, *Fervidibacteria* were observed in higher proportion in most presort populations as compared to the original T_0_ bulk sample. This could be attributed to either favorable growth of this yet uncultured lineage or be a result of preferential cell extraction or cell lysis during freeze–thaw cycles as compared to bulk sample DNA extraction. Alternatively, their increase in relative abundance could have resulted from sample cooling (72 to 55 °C) during transit from the field to the laboratory (4 h).

The Shannon’s diversity indices of the active, sorted fractions were more varied when standardized to the HPG-only control than the presort fractions. This indicates that the activity response among different taxa varies between treatments. For example, incubations with cellobiose or under anoxic conditions led to statistically significantly different diversity indices on average than the HPG-only control incubations (linear mixed effects model, *p* = 0.00349 and *p* = 0.0334, respectively). Cellobiose is a disaccharide of repeating glucose monomers that compose cellulose polymers. Cellobiose can be cleaved by β-1,4 hydrolases into glucose monomers which serve as initial substrate for most central metabolic pathways. The Shannon’s diversity index of cells active with cellobiose amendment was the highest we observed alluding to wide use of cellobiose increasing the evenness of active microbes in these incubations (*p* = 0.00349).

The perimeter of FS5 has a photosynthetic zone (<70 °C; Fig. 1), away from the sampled area, which, together with constant exchange with the atmosphere, serves as a source of O_2_ in the spring (dissolved oxygen levels were 0.909 mg/L at the time of sampling but can vary 1–3 mg/L). Headspace exchange from aerobic (atmospheric air, 21% O_2_) to anoxic conditions (100% N_2_) in the incubations caused the active community to have a significant increase in Shannon’s diversity (*p* = 0.0334) compared to the HPG-only control. This is consistent with the idea that facultative anaerobes persist in the hot spring and their activity varies with oxygen availability.

**Fig. 4:**
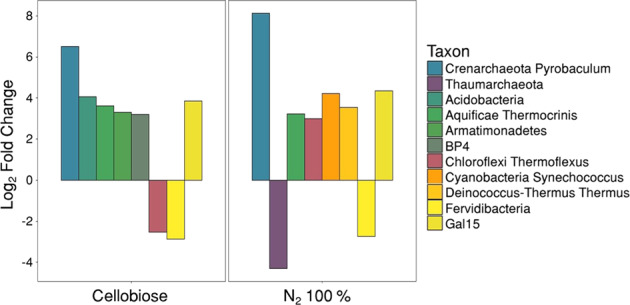
Log2-fold change (LFC) of active taxa in cellobiose substrate amendment and N2 100% headspace condition. Each treatment was compared to the HPG-only control for sorted samples and LFC was calculated. The value for HPG-only was set to 0 and the LFC for each taxon is plotted. Only taxa significantly different from the control are shown (*p* < 0.1). Taxon level represents highest taxonomic resolution for each taxa (Silva 128).

The relative abundance of six taxa increased and two decreased statistically significantly in the active, sorted community in response to cellobiose-amendment based on log_2_-fold change (LFC) when compared to the HPG-only sorted fraction (*p* < 0.10). This suggests that cellobiose has an impact on the activity of several taxa despite the hot spring lacking noticeable large carbon inputs or organic matter that would be degraded regularly in situ. Uncultured phylum *BP4* had an LFC of 3.62 in response to cellobiose amendment (Fig. 4; for a list of all ASVs see Supplementary Table 3). While not much is known about *BP4*, these results could suggest a substrate preference for *BP4* toward cellobiose or its degradation products. In contrast, candidate phylum *Fervidibacteria* decreased in relative abundance in the active fraction of cellobiose amendment with an LFC of −2.88 (Fig. 4). This decrease in *Fervidibacteria* abundance in the active fraction was surprising because this lineage had previously been proposed to have the genomic potential for complete lignocellulose degradation [28]; further, we did not detect a significant response to the addition of cellulose (*p* = 1). These results demonstrate the importance to functionally test genomic predictions under environmentally relevant conditions.

In anoxic incubations, *Thermus* increased (LFC = 3.53, Fig. 4) and represented a large portion of the sorted, translationally active community (Fig. 2). In contrast, a rare member of the *Thaumarchaeota* (averaged relative abundance in the HPG-only sample was 0.4%), whose taxonomic affiliation could not be resolved beyond phylum level, decreased (LFC = −4.31, Fig. 4) in activity in response to anoxic conditions. This is consistent with the observation that all cultured thaumarchaeotes, including thermophilic representatives, have a strictly aerobic lifestyle [29–32].

Sequences related to the genus *Pyrobaculum* exhibited the largest LFC increase in abundance of any taxon detected in the presence of cellobiose (LFC = 6.52) and under anoxic conditions (LFC = 8.12) (Fig. 4). *Pyrobaculum* isolates demonstrate widely varying phenotypic capabilities, including both autotrophic and heterotrophic lifestyles, which can be coupled to H_2_, O_2_, AsO_4_^3−^, S^0^, S_2_O_3_^2−^, SeO_4_^2−^, Fe^3+^, and NO_3_^−^ respiration [33–37]. This metabolic versatility permits *Pyrobaculum* species to occupy geochemically diverse, mildly-acidic to basic (pH > ~4) geothermal environments, including hot springs throughout YNP [38] and likely explains their significant LFC increases in the two treatments.

Cellobiose and anoxic incubations were the only treatments to inform us of community-wide substrate and headspace selectivity with statistical significance. However, organisms belonging to *Thermocrinis* and uncultured phylum *Gal15* increased in relative abundance in both the cellobiose (*p* = 0.0219 and 0.0488, respectively) and anoxic (*p* = 0.0229 and 0.0209, respectively) incubations’ sorted fractions alluding to possible favorable cultivation conditions for these organisms. Other organisms were also detected with large LFCs, though they were not statistically significant when averaged across all three biological replicates. For some samples high variability was observed, emphasizing the need for replication in order to draw biologically meaningful conclusions.

An uncultured member of the *Aigarchaeota* represented a smaller proportion of presort reads (31.4%) after the 2-day incubation than in the original sample (41.9%) (Fig. 2). While *Aigarchaeota* sequences were dominant in the presort fractions, they were not highly abundant in any active fraction (averaged 12.4%). In contrast, the BONCAT-labeled, active fraction of cells were dominated by the uncultured candidate phylum *Fervidibacteria* [39]. *Fervidibacteria* also comprised 48.0% of the total reads across all presorted, bulk communities and the active, sorted cell fraction (Fig. 2). These results could indicate a potential for slow growth or a lack of necessary growth factors for *Aigarchaeota* in our incubations, while *Fervidibacteria* maintained high activity regardless of the amendment. Previously published genomic predictions [26] informed us of possible favorable conditions for *Aigarchaeota*, including acetate and biotin, but none of the tested substrates stimulated a strong activity response. This suggests the importance of unknown community interactions or growth conditions of *Aigarchaeota* that so far have not been replicated in laboratory experiments and highlights the limits of genomic functional predictions and interpretations. *Aigarchaeota* have been proposed to have the genomic potential for both aerobic and anaerobic growth [25] and our experimental results support this hypothesis. Members of *Aigarchaeota* were most abundant in the active fractions of microoxic (20.0%) and anoxic (21.7%) treatments despite not being statistically significant (*p* = 1) (HPG-only = 13.3%). The ability to analyze microbial activity under varying headspace compositions demonstrates that BONCAT-FACS is an approach with great potential for studying cellular responses involving non-assimilatory pathways.

Preliminary experiments determined that 48 h was to be the optimal time for incubation of this sample type to allow for enough HPG incorporation to be detected efficiently on our cell sorter (data not shown). However, this incubation time showed high baseline activity in the HPG-only control making activity variation due to substrate amendment difficult to determine. Extended incubation times with HPG have been shown to not be detrimental to the survival or activity of microorganisms in other sample types [5–7, 9, 11]. However, we posit that proteins related to environmental changes or defensive or stress mechanisms could be responsible for increased protein synthesis. Longer incubation times could select for specific populations and act as pseudo-enrichment allowing “weed” organisms to obscure activity response from hard-to-culture or rare taxa. Despite our limited incubation time, we consistently observed a higher abundance of *Fervidibacteria* populations in presort samples, possibly attributing to higher proportions in sorted, active samples. Alternatively, sampling too early might not provide enough opportunity for organisms to respond to the conditions provided, attributing the BONCAT signal to activity independent of sample treatment, or a general lack of signal. This is a problem with any methodology surveying activity in response to substrate amendment including BONCAT and heavy water SIP [2].

## Conclusion

In this study, the microbial community activity response to substrate amendments and varying oxygen conditions in the headspace was compared to HPG-only incubations that represented close to in situ conditions. This allowed us to determine substrate or oxygen preference of microorganisms without the need for prior cultivation or genomic analysis. The overall composition of the active microbial community did not vary significantly from the HPG-only control (according to Bray–Curtis dissimilarity), but both the Shannon’s diversity indices and the relative abundances of specific microbial populations (LFC) in the active communities were affected by some treatments.

Our benchmark study demonstrates that BONCAT-FACS can be used to identify which taxa in a microbial community are anabolically active and experimentally test genomic predictions about their metabolism in a high-throughput manner and at single cell resolution. Most importantly, BONCAT-FACS is not constrained by the limits of cultivation in order to identify conditions beneficial to the activity of cultured or uncultured archaea and bacteria and can be conducted under as close to in situ conditions as experimentally possible. This information could, in the future, be used for targeted cultivation attempts by informing the design of specific enrichment media or be combined with metagenomic sequencing to provide direct links between cellular function and genetic make-up. With the use of a small set of inexpensive chemicals to label active cells and widely available FACS infrastructure to sort fluorescently labeled cells, BONCAT-FACS can be easily adapted by other labs. Thus, we believe that BONCAT-FACS has strong potential to further progress our understanding of microbial ecophysiology in any natural or human-made system and become a valuable complement to SIP-Raman studies.

## Supplementary information

SI Figure and table legends

SI Figure 1

SI Figure 2

SI Table 1

SI Table 2

SI Table 3

SI Bioinformatics files
