# Supplementary material for: Activity-based cell sorting reveals responses of uncultured archaea and bacteria to substrate amendment
Source: ISME J. 2020 Sep 4;14(11):2851–61. doi: 10.1038/s41396-020-00749-1 (PMC7784905; doi:10.1038/s41396-020-00749-1)
Supplement: Supplementary file 1 — SI Figure and table legends [file 41396_2020_749_MOESM1_ESM.docx]

**Supplementary Figure 1. BONCAT sorting gates. (a)** No-HPG control. **(b)** HPG-only control. First gate is selecting cells based on size. Second gate again selects cells based on size. These two gates restrict larger particles and clumps of cells. The final gate is sorted into collection tubes and selects for BONCAT positive fluorescent cells. The BONCAT gate was drawn conservatively to reduce the number of false positives being collected. The gate was first drawn on the no-HPG sample to collect as few cells as possible. The gate settings were then transferred to all subsequent samples. Values shown next to each gate represent the proportion of events contained within that gate. The red gates are the final gates for each sample that were used for sorting.

**Supplementary Figure 2. BONCAT substrate specificity tests with *E. coli*.** Cultures of *E. coli* were grown in the presence of different substrates and HPG. Relative fluorescence units (RFU) were averaged from three replicates of each condition with error bars representing standard deviation. The cells in the no-HPG condition did not receive HPG during the incubation and thus could not be click stained and the RFU did not measure above the background fluorescence. Cells grown in the presence of sucrose showed only low levels of fluorescence, similar to the HPG-only control, consistent with the observations that *E. coli* is incapable of metabolizing sucrose.

**Supplementary Table 1. Incubation conditions.**  Description of sample names with substrate and HPG amendment. Final concentrations listed, “-“ represents no addition. All samples were incubated in triplicate vials at 74 °C. Unless otherwise indicated, all incubations were done under atmospheric conditions (21% O_2_).

**Supplementary Table 2. Sorter metrics for BONCAT incubations.** Events sorted for each substrate amendment and all replicates listed. Efficiency (%) represents how many positive events were sorted compared to how many positive events had to be aborted and discarded in the FACS prior to sorting. Percent of total events is the number of positive events that comprised the total events before any gating restrictions were applied.

**Supplementary Table 3. Amplicon sequence variants (ASVs) for all taxa shown in sorted samples of Figure 4.** Data processing grouped ASVs for similar taxa into one entry. Shown are the total number of ASVs for each taxon represented as well as the number of ASVs that had 16S rRNA gene sequence counts. Dashed lines represent taxa that were not statistically significant and thus not shown in Figure 4 for that substrate.

**Supplementary files from bioinformatics pipeline in Zip file**

Shannon_index_padjust.csv

Data in this table was used to generate Figure 3. Samples are organized by substrate with the Shannon Index relative to the HPG only samples and p-adjusted value. Values of zero (0) represent samples that were not included in the analysis due to low reads resulting in not enough replicates for the substrate.

organism_removed_from_decontam_0.5prev.csv

List of the contaminant organisms that were removed using Decontam R package with settings set to “method = prevalence” and “threshold = 0.5”.

asv_count_table_post_decontam.csv

Remaining organism read count data for all samples after the contaminate organisms were removed using the Decontam R package.

qiime2_output_asv_count_table.csv

Output from the Qiime2 script containing Feature ID’s and count data for all samples.

qiime2_output_stats.csv

Read counts for all samples throughout the steps of the Qiime2 pipeline. Table contains input reads into the pipeline, resulting filtered reads, denoised reads, merged reads, and final non-chimeric reads.

qiime2_output_taxonomy_table.csv

Output from the Qiime2 script containing feature ID’s and the resulting Taxon ID from the Silva 128 database and associated confidence value.

regression_model.csv

This file contains reads and metadata information for the input in the DESeq2 pipeline of all analyzed samples. Included organisms were the top 42, each containing > 0.01 % relative abundance across the entire data set.

sampledata.csv

Sample metadata describing the condition and attributes of each sample.

taxonomy_table_post_decontam.csv

Resultant taxonomy ID and classification of organisms remaining after processing with Decontam R package.

FeatureID_ASVID_taxa.csv

Conversion table linking Qiime2 feature ID output to ASV ID and taxonomic identification.

FS5_code_ISME.Rmd

Code used for processing Qiime2 data output for contaminate removal, statistical tests, and figure generation.
