# Supplementary figures and images for: Activity-based cell sorting reveals responses of uncultured archaea and bacteria to substrate amendment
Source: ISME J. 2020 Sep 4;14(11):2851–61. doi: 10.1038/s41396-020-00749-1 (PMC7784905; doi:10.1038/s41396-020-00749-1)

Total sorter events

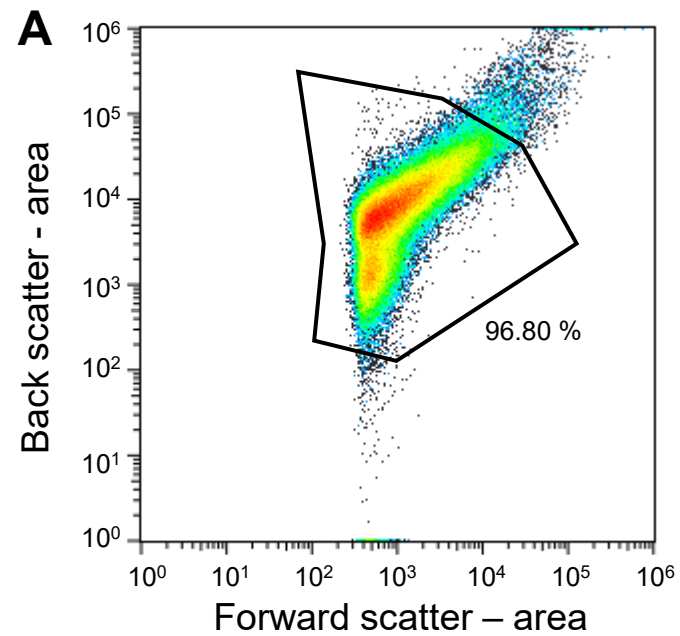

Size restricted events

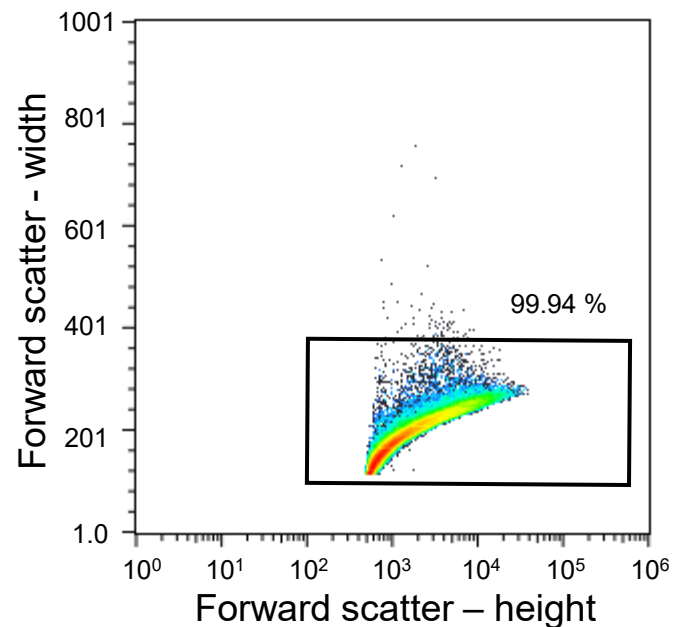

Fluorescent channel events

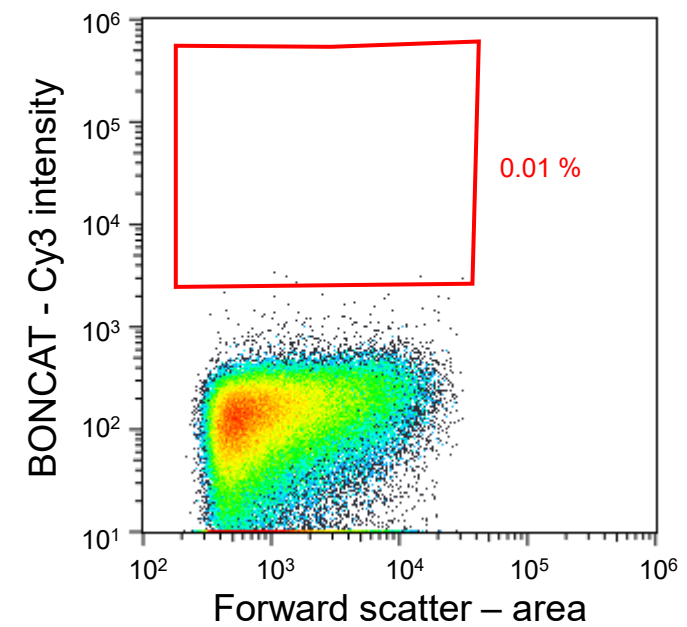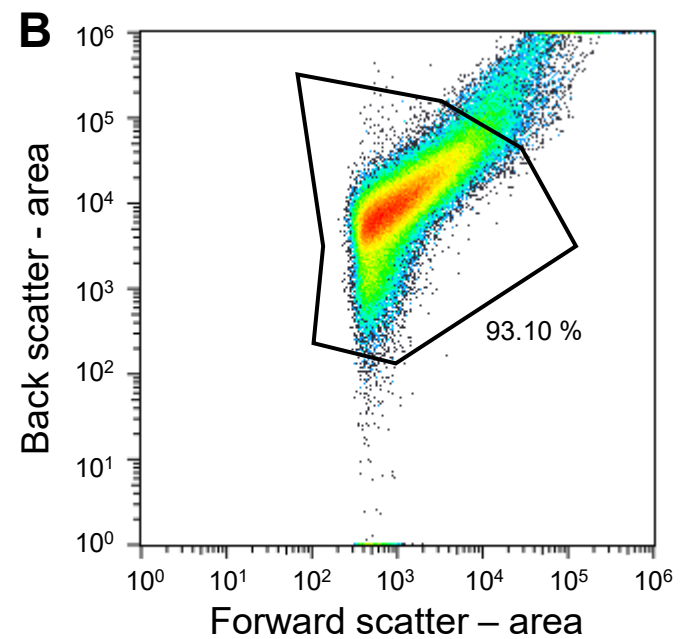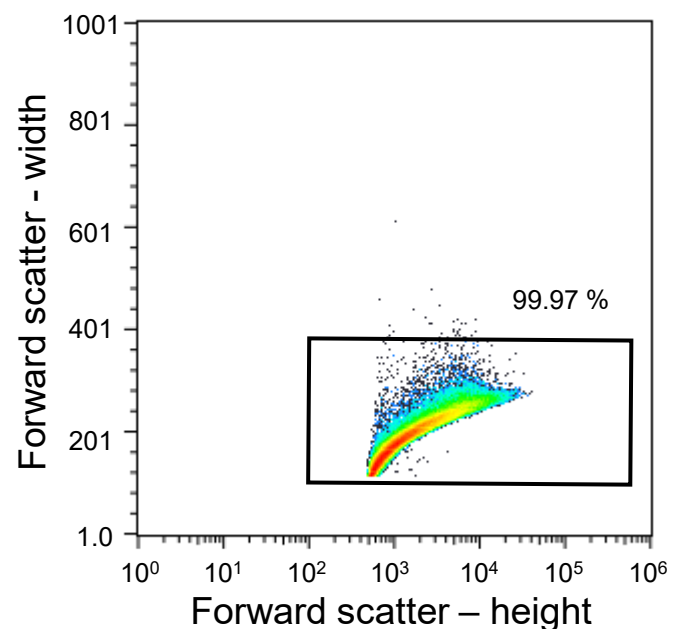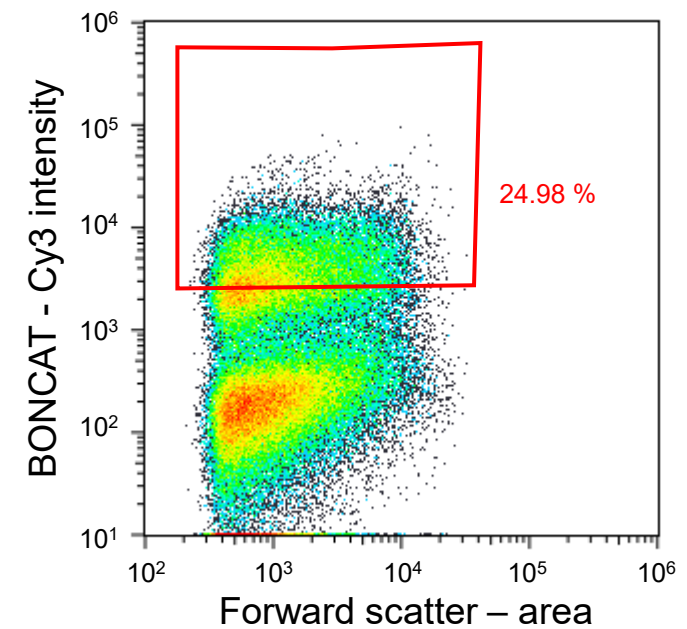

Supplement: Supplementary file 2 — SI Figure 1 [file 41396_2020_749_MOESM2_ESM.pdf]

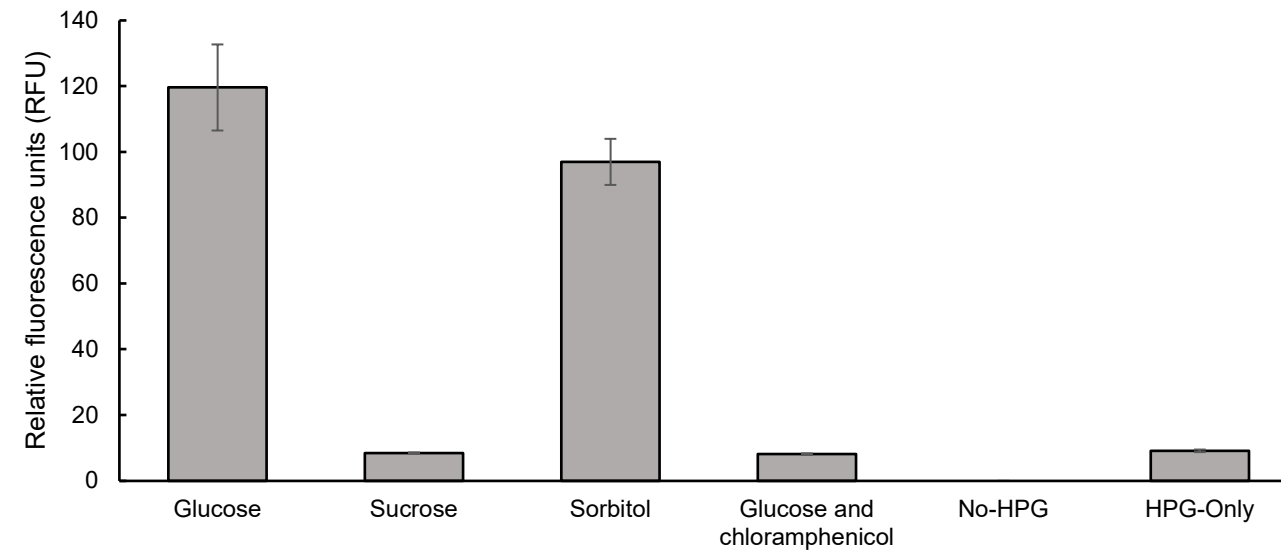

Supplement: Supplementary file 3 — SI Figure 2 [file 41396_2020_749_MOESM3_ESM.pdf]
