# Supplementary material for: Activity-based cell sorting reveals responses of uncultured archaea and bacteria to substrate amendment
Source: ISME J. 2020 Sep 4;14(11):2851–61. doi: 10.1038/s41396-020-00749-1 (PMC7784905; doi:10.1038/s41396-020-00749-1)
Supplement: Supplementary file 4 — SI Table 1 [file 41396_2020_749_MOESM4_ESM.pdf]

Supplementary Table 1. Incubation conditions. Description of sample names with substrate and HPG amendment. Final concentrations listed, "-" represents no addition. All samples were incubated in triplicate vials at 74 °C. Unless otherwise indicated, all incubations were done under atmospheric conditions (21% O_2).

Sample name	Amendment	Substrate concentration	HPG concentration
No-HPG	-	-	-
HPG-only	-	-	50 µM
Acetate	Acetate	10 µM	50 µM
Aspartate	Aspartate	10 µM	50 µM
Biotin	Biotin	10 µM	50 µM
Cellobiose	Cellobiose	10 µM	50 µM
Cellulose	Cellulose	10 µM	50 µM
Glucose	Glucose	10 µM	50 µM
Glycerol	Glycerol	100 µM	50 µM
Glycine	Glycine	10 µM	50 µM
Isoleucine	Isoleucine	10 µM	50 µM
Leucine	Leucine	10 µM	50 µM
NH_4^+ 0.1 mg/L	NH_4^+	5.6 µM	50 µM
NH_4^+ 2 mg/L	NH_4^+	112 µM	50 µM
NH_4^+ 5 mg/L	NH_4^+	280 µM	50 µM
Nitrate	Nitrate	100 µM	50 µM
Nitrite	Nitrite	100 µM	50 µM
Pyruvate	Pyruvate	10 µM	50 µM
Riboflavin	Riboflavin	10 µM	50 µM
Ribose	Ribose	10 µM	50 µM
Serine	Serine	10 µM	50 µM
Thiamine	Thiamine	10 µM	50 µM
Valine	Valine	10 µM	50 µM
Anoxic	N_2 gas	100 % N_2	50 µM
Microoxic	N_2 gas and atmospheric air	90 % N_2, 10 % atmospheric air (2 % O_2)	50 µM
