# Supplementary material for: Activity-based cell sorting reveals responses of uncultured archaea and bacteria to substrate amendment
Source: ISME J. 2020 Sep 4;14(11):2851–61. doi: 10.1038/s41396-020-00749-1 (PMC7784905; doi:10.1038/s41396-020-00749-1)
Supplement: Supplementary file 5 — SI Table 2 [file 41396_2020_749_MOESM5_ESM.pdf]

Supplementary Table 2. Sorter metrics for BONCAT incubations. Events sorted for each substrate amendment and all replicates listed. Efficiency (%) represents how many positive events were sorted compared to how many positive events had to be aborted and discarded in the FACS prior to sorting. Percent of total events is the number of positive events that comprised the total events before any gating restrictions were applied.

Sample name	Replicate 1			Replicate 2			Replicate 3		
	**BONCAT+ events sorted**	**Efficiency (%)**	**Percent of total events**	**BONCAT+ events sorted**	**Efficiency (%)**	**Percent of total events**	**BONCAT+ events sorted**	**Efficiency (%)**	**Percent of total events**
No-HPG	66	97	0.02	43	95	0.01	16	94	0.01
HPG-only	250,000	95	28.76	250,000	92	23.25	191549	97	33.89
Acetate	250,144	95	25.32	250,000	93	28.14	250000	98	64.67
Aspartate	250,000	96	33.32	250,000	93	26.08	207048	99	69.49
Biotin	250,000	95	20.53	250,000	93	30.56	118346	98	59.14
Cellobiose	125,073	93	6.61	199,739	92	10.50	38283	98	16.18
Cellulose	250,000	93	19.52	250,000	93	23.34	162525	98	58.16
Glucose	250,000	95	36.65	250,000	93	32.69	250000	97	56.30
Glycerol	250,000	92	25.44	250,000	93	28.08	250000	97	45.97
Glycine	99,964	93	4.57	250,000	92	16.44	66074	97	19.85
Isoleucine	250,000	92	16.77	179,666	93	10.24	250000	98	60.61
Leucine	250,000	93	25.66	250,000	93	17.52	250000	98	54.54
NH_4^+ 0.1 mg/L	250,000	96	27.37	250,000	91	22.96	250000	97	54.99
NH_4^+ 2 mg/L	250,000	94	21.03	250,000	93	27.88	75177	98	33.22
NH_4^+ 5 mg/L	250,000	94	26.80	250,000	93	27.16	250000	98	51.76
Nitrate	250,000	95	27.33	85,132	96	9.69	250000	97	43.79
Nitrite	175,598	95	24.93	80,164	97	15.69	250000	98	49.55
Pyruvate	250,000	93	25.60	250,000	94	30.93	177582	98	48.67
Riboflavin	250,000	93	20.89	250,000	93	23.46	250000	98	58.44
Ribose	250,000	94	24.03	250,000	93	19.27	250000	98	58.32
Serine	250,000	95	33.29	250,000	94	28.42	250000	97	49.13
Thiamine	250,000	95	33.42	250,000	94	26.88	119730	98	36.01
Valine	250,000	92	20.65	250,000	91	23.02	250000	97	50.00
Anoxic	150,302	86	6.5	125,452	88	4.85	78192	97	16.20
Microoxic	250,000	89	20.38	250,000	91	23.56	250000	97	49.77
