# Supplementary material for: Activity-based cell sorting reveals responses of uncultured archaea and bacteria to substrate amendment
Source: ISME J. 2020 Sep 4;14(11):2851–61. doi: 10.1038/s41396-020-00749-1 (PMC7784905; doi:10.1038/s41396-020-00749-1)
Supplement: Supplementary file 6 — SI Table 3 [file 41396_2020_749_MOESM6_ESM.pdf]

Supplementary Table 3. Amplicon sequence variants (ASVs) for all taxa shown in sorted samples of Figure 4. Data processing grouped ASVs for similar taxa into one entry. Shown are the total number of ASVs for each taxon represented as well as the number of ASVs that had 16S rRNA gene sequence counts. Dashed lines represent taxa that were not statistically significant and thus not shown in Figure 4 for that substrate.

Taxon	Cellobiose ASVs with sequencing count	N_2 100 % ASVs with sequencing count	Total ASVs
Crenarchaeota Pyrobaculum	1	3	37
Thaumarchaeota	-	1	4
Acidobacteria	4	-	5
Armatimonadetes	9	-	36
Aquificae Thermocrinis	17	14	58
BP4	13	-	34
Chloroflexi Thermoflexus	3	5	32
Cyanobacteria Synechococcus	-	2	29
Deinococcus-Thermus Thermus	-	27	127
Fervidibacteria	19	10	128
Gal15	5	3	13
